# Impact of kinship matrices on genetic gain and inbreeding with optimum contribution selection in a genomic dairy cattle breeding program

**DOI:** 10.1186/s12711-023-00826-x

**Published:** 2023-07-17

**Authors:** Egill Gautason, Goutam Sahana, Bernt Guldbrandtsen, Peer Berg

**Affiliations:** 1 Center for Quantitative Genetics and Genomics, Aarhus University, 8000 Aarhus, Denmark; 2 Faculty of Agricultural Sciences, Agricultural University of Iceland, 311 Borgarbyggð, Iceland; 3 Department of Veterinary and Animal Sciences, University of Copenhagen, 1870 Frederiksberg C, Denmark; 4 Faculty of Life Sciences, Norwegian University of Life Sciences, 1430 Ås, Norway

## Abstract

**Background:**

Genomic selection has increased genetic gain in dairy cattle, but in some cases it has resulted in higher inbreeding rates. Therefore, there is need for research on efficient management of inbreeding in genomically-selected dairy cattle populations, especially for local breeds with a small population size. Optimum contribution selection (OCS) minimizes the increase in average kinship while it maximizes genetic gain. However, there is no consensus on how to construct the kinship matrix used for OCS and whether it should be based on pedigree or genomic information. VanRaden’s method 1 (VR1) is a genomic relationship matrix in which centered genotype scores are scaled with the sum of 2*p*(1-*p*) where *p* is the reference allele frequency at each locus, and VanRaden’s method 2 (VR2) scales each locus with 2*p*(1-*p*), thereby giving greater weight to loci with a low minor allele frequency. We compared the effects of nine kinship matrices on genetic gain, kinship, inbreeding, genetic diversity, and minor allele frequency when applying OCS in a simulated small dairy cattle population. We used VR1 and VR2, each using base animals, all genotyped animals, and the current generation of animals to compute reference allele frequencies. We also set the reference allele frequencies to 0.5 for VR1 and the pedigree-based relationship matrix. We constrained OCS to select a fixed number of sires per generation for all scenarios. Efficiency of the different matrices were compared by calculating the rate of genetic gain for a given rate of increase in average kinship.

**Results:**

We found that: (i) genomic relationships were more efficient than pedigree-based relationships at managing inbreeding, (ii) reference allele frequencies computed from base animals were more efficient compared to reference allele frequencies computed from recent animals, and (iii) VR1 was slightly more efficient than VR2, but the difference was not statistically significant.

**Conclusions:**

Using genomic relationships for OCS realizes more genetic gain for a given amount of kinship and inbreeding than using pedigree relationships when the number of sires is fixed. For a small genomic dairy cattle breeding program, we recommend that the implementation of OCS uses VR1 with reference allele frequencies estimated either from base animals or old genotyped animals.

## Background

Genetic diversity must be preserved to achieve genetic gain in future generations, and inbreeding must be managed to avoid negative effects on traits. Genetic diversity within livestock breeds is preserved and inbreeding is controlled by managing effective population size, by controlling the rate of increase in average kinship. In recent years, the implementation of genomic breeding has increased the threat of eroding the genetic diversity in dairy cattle in at least two ways. First, some genomically-selected populations have shown increased inbreeding rates and smaller effective population sizes [1–4]. Second, small and local breeds have become increasingly less competitive because, in general, they have not implemented genomic selection. Simulations have shown that small dairy cattle populations can also benefit economically from genomic selection [5, 6]. It is important that implementation of a genomic program considers how to manage genetic diversity and inbreeding. To compare different breeding programs, the efficiency of inbreeding management can be considered as the rate of genetic gain at the same rate of increase in average inbreeding [7].

Optimum contribution selection (OCS) maximizes genetic gain while it restrains inbreeding by managing long-term genetic contributions [8]. Several studies have shown that OCS can achieve more genetic gain than truncation selection [8–13]. However, the use of OCS has not been widely adopted in dairy cattle breeding due to the decentralized structure of such breeding programs [12]. Icelandic Cattle is a dairy cattle population with a centralized structure, for which the most important selection decisions are taken by a committee of farmers and specialists, which makes it ideal for the application of OCS. Therefore, our aim was to study the use of OCS using Icelandic Cattle as a model. Icelandic Cattle is a local breed that has most likely been almost completely isolated for over 1000 years and is genomically distinct from other European populations [14], and genomic inbreeding estimates do not indicate severe historical inbreeding [15]. A study of genomic prediction accuracies indicated that genomic selection is a realistic option [16] and a genomic breeding program is currently being implemented. It is a closed population with no import of dairy cattle genetics being currently allowed. For such a closed, local population with a high conservation value, it is especially important to preserve its genetic diversity to ensure long-term genetic gain.

The cost of raising bulls and collecting semen for artificial insemination (AI) is a major part of the costs for dairy cattle breeding programs [17], especially for small, local populations. In a real breeding program, OCS recommendations can rarely be entirely followed due to logistical and biological restrictions [18]. Without constraints, OCS can suggest a number of matings that is not realistic for a real population, for example by suggesting varying numbers of selected bulls per selection cycle, or an unreasonably large number of bulls. In a real situation, the breeding program will be constrained by staff, housing and funds to buy, house, feed and collect semen from AI bulls. These logistical constraints limit the degree to which OCS recommendations can be followed. We argue that dairy cattle breeding schemes, for populations such as Icelandic Cattle, should be compared using the same number of bulls selected per selection cycle. For a population that does not aim at exporting semen, a cost-effective way is to collect semen in roughly equal amounts of semen doses from each bull.

Pedigree relationships have been used to implement OCS (POCS). Genomic OCS (GOCS) is possible by using a genomic relationship matrix (GRM) instead of the pedigree relationship matrix (numerator relationship matrix). Sonesson et al. [19] and Henryon et al. [20] studied the use of GOCS and POCS in populations with genomic predictions. Sonesson et al. [19] concluded that GOCS was preferable to POCS, but Henryon et al. [20] found that POCS allowed for more genetic gain than GOCS. Henryon et al. [20] emphasized the need for more research on the approach to estimate genomic relationships for controlling inbreeding but recommended the use of POCS until more was known. They also argued that POCS allowed for larger changes in allele frequency at quantitative trait loci (QTL) than GOCS and this made POCS more efficient than GOCS. A way to allow for larger changes in allele frequency at QTL with GOCS is to update reference allele frequencies (RAF) of marker loci when constructing the GRM, for example using the current generation or all genotyped animals. Doing this with GOCS will restrain changes in allele frequency relative to the current population rather than the accumulated change over earlier generations, as noted by Meuwissen et al. [21]. Different GRM can be used for GOCS, and it is important to identify the most efficient one. We considered two GRM, VanRaden’s methods 1 and 2 [22]. These methods are among the most commonly used methods for setting-up GRM in animal breeding. VanRaden’s method 1 (VR1) scales the cross-product of centered genotype scores by $\sum 2p(1-p)$, where $p$ is the RAF at each marker locus. VanRaden’s method 2 (VR2) weights each marker by the reciprocal of $2p(1-p)$, thereby giving greater weight than VR1 to markers with a low minor allele frequency (MAF). The different weighting of the loci results in different estimates of relationships and thereby different results of GOCS. Meuwissen et al. [21] compared the use of GRM and pedigree relationships in an OCS scheme and partitioned inbreeding into two components: (i) increase in homozygosity, ${F}_{hom}$ and (ii) increase in drift, ${F}_{drift}$. They found that the type of relationship matrix influenced increase in ${F}_{hom}$ and ${F}_{drift}$ differently, with some matrices resulting in higher values of ${F}_{hom}$ or ${F}_{drift}$, and some resulting in roughly equal ${F}_{hom}$ and ${F}_{drift}$. They concluded that the choice of a GRM for OCS should depend on the objective of the inbreeding management, i.e. whether it is to minimize drift or minimize homozygosity.

The aim of this study was to compare the performance of nine kinship matrices for use with OCS: the numerator relationship matrix and two types of GRM, VR1 and VR2 using different RAF in a simulated dairy cattle population undergoing genomic selection. We compared (i) rate of increase in average kinship, (ii) rate of genetic gain, (iii) rate of loss of additive genetic variance and additive genic variance, (iv) rate of average inbreeding according to drift and homozygosity, and (v) change in MAF at QTL and neutral loci.

## Methods

The simulations were modelled after the breeding program of the Icelandic Cattle population. Simulations were performed using QMSim [23] for simulating a base population and the R [24] package MoBPS (Modular Breeding Program Simulator) was used for breeding program simulations [25]. We used GMATRIX [26] to construct GRM and we used EVA [27] to optimize genetic contributions of selection candidates. The DMU software package [28] was used to predict breeding values.

### Historical population

We used QMSim to simulate a historical population in which the linkage disequilibrium (LD) is similar to that in Icelandic Cattle [15]. We simulated ten replicates of the historical population, each encompassing all possible scenarios. The population size was 2000 for 2000 generations. Then, the population size was reduced to 200 over 100 generations, and then increased over another 100 generations to 1000. The sex ratio was 1 to 1 in the historical population. In the last generation, 6000 females and 6000 males were generated to form the base population of the simulation, generation zero. The genome consisted of 29 chromosomes that were 100 cM long, each with 1800 evenly spaced biallelic loci. The marker allele frequency was 0.5 at each locus in the first historical generation and recurrent mutations were simulated with a mutation rate of 2 × 10^–5^ per allele per generation. No new mutations were simulated but the allele state was altered between the two alternate alleles. Mutations were simulated only for the historical population. Genotype data of the historical population were converted into PLINK [29] *ped* format and were loaded into the MoBPS R package. We randomly selected 3000 segregating loci as QTL, and 3000 loci were selected as neutral non-marker loci. We did not apply any MAF criteria to select QTL and neutral non-marker loci. These 6000 neutral loci and QTL were used neither to construct GRM nor to predict breeding values. Marker loci were selected from the remaining segregating loci. To achieve a distribution of MAF that resembled commercial single nucleotide polymorphism (SNP) chips, we discarded SNPs with a low MAF, because commercial SNP chips have a MAF distribution that is closer to a uniform distribution than the distribution of MAF for neutral alleles. We used the following rules: if a locus had a MAF lower than 0.01, there was a 50% probability of discarding it. If a locus had a MAF lower than 0.02 but higher then 0.01, there was a 20% probability of discarding the loci. After this filtration, all remaining loci were used as marker loci. Thus, there were three types of loci: (i) marker loci, (ii) QTL, and (iii) neutral non-marker loci. The number of marker loci differed slightly between replicates, ranging from 39,521 to 39,785 for ten replicates. Following this, we assigned effects to QTL by drawing them from a gamma distribution with a shape parameter of 0.4 [30] and a scale parameter of 1.66 [31]. We simulated a trait that was recorded only on females with a heritability of 0.4 in the base population and QTL effects were additive. To simulate phenotypes, we used a constant residual variance. Therefore, the heritability of the trait varied with the additive genetic variance. The numbers of recombinations were sampled from a Poisson distribution with an expectation of 1 per Morgan, and locations were random across the genome.

### Breeding program structure

We simulated one generation of random selection and four generations of pedigree-based best linear unbiased prediction (PBLUP) selection before simulating 15 generations of genomic selection. Generations were discrete and no selection was applied on the female side. In each generation, 6000 male and 6000 female offspring were generated. The number of females was selected to reflect the number of females contributing to breeding in the Icelandic Cattle population. To reach this figure, we counted the number of herds that contributed bull calves to the Icelandic progeny testing program from 2014 to 2018, and divided it by the total number of herds in the period (128/558). This proportion was multiplied by the number of breeding females in the population (26,000), which gave approximately 6000. Genomic evaluations were used to predict breeding values, either using a genomic relationship matrix [genomic BLUP (GBLUP)] or a combined genomic and pedigree relationship matrix [single step genomic BLUP (ssGBLUP)]. In the first three generations of genomic selection, ssGBLUP was used to predict breeding values and in the subsequent 12 generations, GBLUP was used to reduce computations because the ssGBLUP evaluation is computationally time-consuming when the number of genotyped animals is large. We used the genotypes of eight generations of animals with data for the genomic evaluations.

#### Pedigree-based selection—generations 0–5

Selection in the first generation was random, followed by four generations of PBLUP based truncation selection. In each generation, 120 males were selected, randomly mated to females, and used equally. Phenotypes were available for females when selection was performed. To build a reference population for genomic prediction, selected sires in generations zero to five were genotyped and all females in generation five were genotyped.

#### Genomic selection—generations 6–20

In each generation, the parent average ($PA$) predicted breeding value of males was computed as $PA=\frac{{GEBV}_{sire}+{GEBV}_{dam}}{2}$. The 2000 males with the highest $PA$ were genotyped and the other 4000 were not considered for selection. All 6000 females were genotyped. The dams of the selection candidates had phenotypes at the selection stage. The breeding values of the candidates were then predicted using GBLUP or ssGBLUP, as described below. OCS was implemented by constructing a *pseudofemale* and mating her to males while restricting the OCS computations to select 40 males, as described below. The 40 selected males were then randomly mated to females with an equal number of matings per bull.

### Breeding value prediction

The model for predicting breeding values included only the additive genetic animal effect, an intercept and a residual:

$$\mathbf{y}=\boldsymbol{1}\upmu +\mathbf{Z}\mathbf{a}+\mathbf{e},$$

where $\mathbf{y}$ is a vector of animal phenotypes, $\boldsymbol{1}$ is a vector of ones, $\upmu$ is the phenotypic mean, $\mathbf{a}$ is a vector of predicted breeding values or genomic estimated breeding values (GEBV). For PBLUP, all animal phenotypes were used in $\mathbf{y}$. For genomic selection, phenotypes of all genotyped animals were used in $\mathbf{y}$. In later generations, eight generations of animal phenotypes were used. For PBLUP, $\mathbf{a}$ followed a normal distribution $N(\boldsymbol{0}, \mathbf{A}{\sigma }_{A}^{2})$, where $\mathbf{A}$ is the numerator relationship matrix, $\boldsymbol{0}$ a vector of zeroes, and ${\sigma }_{A}^{2}$ the additive genetic variance (variance of true breeding values in generation zero). For ssGBLUP, $\mathbf{a}$ followed the distribution $N(\boldsymbol{0},\mathbf{H}{\sigma }_{A}^{2})$, where $\mathbf{H}$ is a combined pedigree and genomic relationship matrix [32] for which the GRM was computed using VR1 (see below). For GBLUP, $\mathbf{a}$ followed $N(\boldsymbol{0},\mathbf{G}{\sigma }_{A}^{2})$, where $\mathbf{G}$ is the GRM computed using VR1. Different numbers of animals were evaluated per generation. For ssGBLUP, 6000 males and 6000 females were evaluated per generation, but for GBLUP, 2000 males and 6000 females were evaluated. Thus, the matrices $\mathbf{H}$ and $\mathbf{G}$, and corresponding vector $\mathbf{y}$ and matrices $\mathbf{Z}$ and $\mathbf{I}$ had different sizes in ssGBLUP and GBLUP. $\mathbf{Z}$ is a design matrix that relates records to random genetic effects. $\mathbf{e}$ is a vector of random residuals following $N(\boldsymbol{0}, \mathbf{I}{\sigma }_{e}^{2})$, where ${\sigma }_{e}^{2}$ is the residual variance.

### Relationship matrices

We used the first (VR1) and second (VR2) methods of VanRaden [22] to construct GRM using GMATRIX [26]. VR1 was computed as follows:

$$\mathbf{G}=\frac{\mathbf{Z}\mathbf{Z}\mathbf{^{\prime}}}{2\sum_{j=1}^{m}{p}_{j}(1-{p}_{j})},$$

where $\mathbf{Z}=\mathbf{M}-\mathbf{P}$, where $\mathbf{M}$ is the genotypic matrix for genotyped animals. The rows of $\mathbf{M}$ are genotypes with values 2 or 0 for homozygotes and 1 for heterozygotes. The columns of $\mathbf{M}$ correspond to the marker loci. $\mathbf{P}$ is a matrix in which all elements in the $j$th column were $2{p}_{j}$ with ${p}_{j}$ being the frequency of the allele that is counted in $\mathbf{M}$ (the RAF) at locus $j$, and $m$ is the number of marker loci.

VR2 was similarly computed as:

$\mathbf{G}=\mathbf{Z}\mathbf{D}\mathbf{Z}\boldsymbol{^{\prime}}$, where $\mathbf{D}$ is a diagonal matrix with elements ${d}_{jj}=\frac{1}{m(2{p}_{j}\left(1-{p}_{j}\right))}$, $m$ and ${p}_{j}$ being defined as above. We did not apply any MAF filtering when constructing the GRM. For each of these methods, we used different approaches to estimate RAF. Genomic relationship matrices **VR1 Base**, **VR2 Base**, **VR1 All**, **VR2 All**, **VR1 Current** and **VR2 Current** were computed using VR1 and VR2, and RAF were computed using (i) animals in generation one (Base); (ii) all genotyped animals (All); and (iii) genotyped animals in the current generation (Current). In addition, we used VR1 with a RAF of 0.5 for all markers, i.e. **VR1 0.5**. Using a value of 0.5 as RAF with OCS contributes in maintaining a maximum heterozygosity rather than minimizing drift. We also used VR1 with RAF estimated from the selected and genotyped bulls in generations zero to three, **VR1 Old**. This scenario represented information that might be available to breeding programs by genotyping semen samples from old insemination bulls. Lastly, we used the numerator relationship matrix, **Pedigree**. These nine matrices represent the different studied scenarios. Eight scenarios used GOCS and one scenario used POCS. The different matrices are based on three different measures of diversity; **Pedigree** is based on probabilities of identity-by-descent, **VR1 0.5** is based on heterozygosity, and the other genomic matrices are based on changes in allele frequency. To reduce simulation errors, we used the same base population for all scenarios in each replicate across scenarios.

### Optimum contribution selection

Bulls were selected according to OCS based on their GEBV and one of the relationship matrices described above. To reduce computations, only 1000 genotyped males with GEBV above the median were considered for selection. OCS was implemented with constraints to select a fixed number of sires. We constructed a pseudofemale based on all the females. The pseudofemale had a relationship with herself that was equal to the mean relationship among females, including self-relationships, and a relationship with each bull that was equal to the mean relationship of all female candidates to that bull. The pseudofemale was assigned 40 matings, and each of the 1000 bulls was allowed a maximum of one mating. This simplification reduced computation time drastically and is a valid approximation when there is no selection on the dam side. The genetic contributions were optimized to achieve the target rate of inbreeding, while maximizing genetic gain. This implementation means that the contribution of each candidate was fixed and thus the optimized parameter was whether the bull was selected or not. Genetic contributions $\mathbf{c}$ were optimized in each generation to maximize the genetic level in the offspring generation, $\mathrm{G}$:

$$\mathrm{G}={\mathbf{c}\mathbf{^{\prime}}}\widehat{\mathbf{a}},$$

where $\widehat{\mathbf{a}}$ is a vector of GEBV, given the constraint:

$$\mathbf{c}\mathbf{^{\prime}}\mathbf{R}\mathbf{c}\le \mathrm{C}$$

where $\mathbf{R}$ is a kinship matrix, the target average kinship $\mathrm{C}$ was set such that the rate of increase was 0.005 for a target effective population size of 100, and the constraint that the sum of contributions of males and females each equaled ½ [8]. To achieve a rate of increase in average kinship that was equal to the target increase, we used a kinship matrix $\mathbf{R}$ that included both genotyped and non-genotyped bulls. Therefore, the bulls whose genotype information was not used in the simulated breeding program were included in $\mathbf{R}$ for the OCS computations but they were not candidates for selection. We did this so that the rate of increase in average kinship was computed relative to the whole population, and not only relative to the genotyped animals. This would not be possible for GOCS in practice because genotype information for these bulls would not be available. However, it was necessary to ensure that the rate of increase in average kinship was close to the target of 0.005. The 40 selected bulls were then randomly mated to cows and equally used. The number of matings assigned to each bull candidate was equal to: $\frac{Number \,of \,females}{Number \,of \,males \,selected}=150$. Each cow was randomly mated to two bulls and had exactly two calves in each generation. The sex of each calf was random and 6000 male and 6000 female calves were simulated in each generation.

### Statistical analysis

In each generation, we computed mean true breeding value, mean kinship, additive genetic variance (${\sigma }_{A}^{2}$), additive genic variance (${\sigma }_{G}^{2}$), number of polymorphic QTL, neutral loci, and marker loci, and MAF at QTL and neutral loci. The true breeding value of each animal was computed as the sum of the genotypic effects across all loci in each animal. The pairwise kinship ($f$) was estimated in MoBPS with the function, *kinship.emp.fast*, which uses recombination points to compute the proportion of chromosome segments between two individuals that are identical-by-descent when a haplotype is drawn at random from each individual. The mean kinship was estimated using 360,000 randomly selected pairwise relationships, out of the total of 71,994,000 pairwise relationships for each cohort of 12,000 animals. Additive genetic variance was the variance of true breeding values in each generation and additive genic variance was computed as the sum of QTL additive genetic variance at each locus as ${\sigma }_{G}^{2}=\sum p(1-p){\alpha }^{2}$, where $\alpha$ is the QTL effect, using the function get.qtl.variances() in MoBPS. The main difference between these two estimates of additive genetic variance is that ${\sigma }_{G}^{2}$ assumes linkage equilibrium and Hardy–Weinberg proportions [44]. The Bulmer effect reduces the additive genetic variance by inducing LD and thereby reduces ${\sigma }_{A}^{2}$ but does not affect ${\sigma }_{G}^{2}$. Mating of relatives increases ${\sigma }_{A}^{2}$ but does not affect ${\sigma }_{G}^{2}$. To quantify the levels of drift and homozygosity in the population, we computed two inbreeding coefficients, ${F}_{drift}$ and ${F}_{hom}$ [21] using allele frequencies in the fifth generation of the simulation as base. ${F}_{hom}$ is a measure of the current expected homozygosity relative to the reference population, and thus reflects loss of heterozygosity. ${F}_{hom}$ was computed as:

1 $${F}_{hom}=\frac{1}{m}\sum_{k=1}^{m}\frac{2{p}_{t,k}\left(1-{p}_{t,k}\right)}{2{p}_{5,k}\left(1-{p}_{5,k}\right)},$$

where ${p}_{t,k}$ is the allele frequency of locus $k$ at generation $t$, and $m$ is the number of loci. Drift can be measured as the squared deviation of allele frequencies from an initial state and “scaled by the expected value for complete random inbreeding” [21]. Generation five was used as a reference because it corresponded to the start of genomic selection. ${F}_{drift}$ was computed as:

2 $${F}_{drift}=\frac{1}{m}\sum_{k=1}^{m}\frac{{\left({p}_{t,k}-{p}_{5,k}\right)}^{2}}{{p}_{5,k}\left(1-{p}_{5,k}\right)}.$$

These statistics, ${F}_{hom}$ and ${F}_{drift}$, were computed separately for QTL, neutral loci and marker loci. To avoid the large effect of very low MAF alleles, we only computed ${F}_{hom}$ and ${F}_{drift}$ for loci with a MAF > 0.001 in the fifth generation. Below, $f$, ${F}_{drift}$ and ${F}_{hom}$ always refer to population averages, and $\Delta f$, ${\Delta F}_{drift}$ and ${\Delta F}_{hom}$ refer to rates of increase in the average of these parameters from the fifth generation. We plotted genetic gain, using the R package ggplot2 [33], as a function of $f$, ${F}_{drift}$ and ${F}_{hom}$ to compare the efficiency of different scenarios at achieving genetic gain. We used regression coefficients obtained from a linear model to compare the scenarios. The model corrected the dependent variable for the effect of replicate and regressed the dependent variable on generation for each scenario. We used the following fixed effects model, which was implemented using the *lm()* function in R, for generations 5 to 20:

3 $${y}_{klm}={\beta }_{k}+{\beta }_{l}{X}_{lm}+{e}_{klm},$$

where the dependent variable $y$ is the average true breeding value *G*, additive genetic variance (${\sigma }_{A}^{2}$), additive genic variance (${\sigma }_{G}^{2}$), $-log(1-f)$, $-log(1-{F}_{drift})$, or $-log(1-{F}_{hom})$, of the $k$ th replicate in the $l$ th scenario in the $m$ th generation, ${\beta }_{k}$ is the intercept of the $k$ th replicate and ${\beta }_{l}$ is the regression coefficient of *y* on $X$, where $X$ is the generation number 5 to 20, and $e$ is the random residual. The regression coefficient ${\widehat{\beta }}_{l}$ was used to compare scenarios, and represents the unit change in the average of these parameters per generation, denoted with $\Delta$. Average true breeding value was expressed as units of additive genetic standard deviations (${\sigma }_{A}$) in generation zero. Additive genetic variance and additive genic variance were expressed in percentages relative to their values in generation 5. We used a t-test to test pairwise significance of differences of the linear regression coefficients, comparing all pairs of scenarios. We used a Bonferroni correction to correct for multiple testing, dividing the P-value obtained by the number of pairwise comparisons (9 × 8/2 = 36).

## Results

Rate of increase in average kinship ($\Delta f$), rate of genetic gain ($\Delta G$), rate of loss of additive genetic variance (${\Delta \sigma }_{A}^{2}$), rate of loss of additive genic variance ($\Delta {\sigma }_{G}^{2}$), and MAF at QTL and neutral loci are in Table 1. Table 1 also shows $\Delta f$, $\Delta G$, $\Delta {\sigma }_{G}^{2}$, and ${\Delta \sigma }_{A}^{2}$ relative to **Pedigree**. The GOCS scenarios using **Base**, **Old**, and **All** as RAF resulted in less $\Delta f$, $\Delta {\sigma }_{G}^{2}$, and ${\Delta \sigma }_{A}^{2}$ than **Pedigree**, but only slightly lower $\Delta G$. **VR1 Base** achieved 96% of the $\Delta G$ that was achieved in **Pedigree** at a $\Delta f$ that was equal to 67% of that of **Pedigree**.

**Table 1:** Rate of increase in average kinship, genetic gain, loss of genetic variance, and average MAF

Scenario	$\Delta f$ (%)	$\Delta G$	$\Delta {\sigma }_{A}^{2}$ (%)	$\Delta {\sigma }_{G}^{2}$ (%)	Relative to Pedigree*				MAF	
					$\Delta f$	$\Delta G$	$\Delta {\sigma }_{A}^{2}$	$\Delta {\sigma }_{G}^{2}$	QTL	Neutral
VR1 Base	0.500^a^	0.863^a^	− 2.5^a^	− 2.4^a^	0.67	0.96	0.80	0.91	0.201	0.209
VR1 Old	0.510^a^	0.865^a^	− 2.6^b^	− 2.5^a^	0.68	0.96	0.84	0.92	0.201	0.210
VR2 Base	0.514^a^	0.865^a^	− 2.5^a^	− 2.4^a^	0.69	0.96	0.81	0.91	0.198	0.207
VR1 All	0.606^b^	0.892^b^	− 2.9^c^	− 2.6^b^	0.81	0.99	0.94	0.96	0.195	0.206
VR2 All	0.646^c^	0.897^bc^	− 3.0^c^	− 2.6^b^	0.87	1.00	0.97	0.97	0.193	0.201
VR1 0.5	0.702^e^	0.896^bc^	− 2.9^c^	− 2.6^b^	0.94	1.00	0.95	0.96	0.198	0.208
VR1 Current	0.785^d^	0.903^d^	− 3.1^d^	− 2.7^c^	1.05	1.00	1.00	1.01	0.185	0.194
VR2 Current	0.785^d^	0.905^d^	− 3.1^d^	− 2.7^c^	1.05	1.01	1.00	1.02	0.184	0.193
Pedigree	0.746^f^	0.900^c^	− 3.1^d^	− 2.7^c^	1.00	1.00	1.00	1.00	0.186	0.194

*Values are proportional to those in Pedigree

Values in the same column with different superscript letters are significantly different (P < 0.05) while values in the same column that share a superscript letter are not significantly different

$\Delta f$: rate of increase of average kinship in percentages; $\Delta G$: rate of genetic gain expressed in units of additive genetic standard deviations; $\Delta {\sigma }_{A}^{2}$: rate of loss of additive genetic variance in percentages relative to generation five; $\Delta {\sigma }_{G}^{2}$: rate of loss of additive genic variance in percentages relative to generation five; MAF: average minor allele frequency at QTL and neutral loci

The choice of RAF had a substantial effect. With more recent RAF, from **Base** to **Current**, both $\Delta f$ and $\Delta G$ increased. **VR1 Base** had both the lowest $\Delta f$ and the lowest $\Delta G$. **VR1 Current** and **VR2 Current** had both the highest $\Delta f$ and the highest $\Delta G$. **VR1 Base** resulted in $\Delta f$, $\Delta {\sigma }_{G}^{2}$, and ${\Delta \sigma }_{A}^{2}$ that were 0.29, 0.6 and 0.3 percentage points lower per generation than **VR1 Current**, while the difference in $\Delta G$ was only 0.04 ${\sigma }_{A}$ per generation.

There was no significant difference in $\Delta f$, $\Delta G$, $\Delta {\sigma }_{G}^{2}$, and ${\Delta \sigma }_{A}^{2}$ between VR1 and VR2 when they were compared using the same RAF, except for $\Delta f$ when **All** was used (P < 0.05). However, VR1 resulted numerically in less $\Delta f$ and less $\Delta G$ than VR2 when **Base** and **All** were used as RAF. Minor allele frequency followed the same trend as $\Delta f$ across scenarios. With more recent RAF, average MAF was lower at QTL and neutral loci, but the genetic gain was highest.

Table 2 shows ${\Delta F}_{drift}$, ${\Delta F}_{hom}$_*,*_, and ${F}_{hom}-{F}_{drift}$ in the last generation, at neutral loci, QTL, and marker loci. ${\Delta F}_{drift}$ was higher than ${\Delta F}_{hom}$ in all scenarios. ${\Delta F}_{hom}$ at neutral loci and markers was lower than $\Delta f$ in all scenarios. VR1 tended to result in lower ${\Delta F}_{drift}$ and lower ${\Delta F}_{hom}$ than VR2 but the differences were mostly not statistically significant. At the end of the simulation, average ${F}_{drift}$ was higher than average ${F}_{hom}$ in all scenarios. Levels of ${F}_{drift}$ and ${F}_{hom}$ were most similar in **VR2 Base** and most dissimilar in **VR1 0.5**.

**Table 2:** Rates of inbreeding based on drift and homozygosity at neutral loci, QTL and marker loci

Scenario	${\Delta F}_{drift}$ (%)			${\Delta F}_{hom}$ (%)			${F}_{hom}-{F}_{drift}$ (%)		
	Neutral	QTL	Markers	Neutral	QTL	Markers	Neutral	QTL	Markers
VR1 Base	0.656^a^	0.945^a^	0.654^a^	0.427^a^	0.524^a^	0.414^a^	− 1.8	− 4.4	− 2.4
VR1 Old	0.640^a^	0.926^a^	0.639^a^	0.453^a^	0.564^ad^	0.464^b^	− 1.3	− 3.7	− 1.1
VR2 Base	0.666^a^	0.955^a^	0.663^a^	0.463^a^	0.605^b^	0.484^b^	− 1.1	− 2.9	− 0.7
VR1 All	0.750^b^	1.053^b^	0.749^b^	0.546^b^	0.702^c^	0.557^c^	− 2.4	− 4.2	− 2.1
VR2 All	0.795^c^	1.108^c^	0.797^c^	0.641^c^	0.721^c^	0.612^d^	− 1.2	− 4.8	− 1.8
VR1 0.5	0.881^d^	1.205^d^	0.890^d^	0.504^d^	0.591^bd^	0.485^b^	− 7.0	− 10.4	− 7.6
VR1 Current	0.950^e^	1.277^e^	0.954^e^	0.676^ cd^	0.849^ef^	0.729^e^	− 2.4	− 4.1	− 2.2
VR2 Current	0.945^e^	1.258^ef^	0.944^e^	0.734^e^	0.877^e^	0.742^e^	− 3.4	− 5.7	− 2.7
Pedigree	0.928^e^	1.244^f^	0.922^f^	0.683^d^	0.835^f^	0.695^f^	− 3.2	− 5.0	− 2.5

Values are in percentages. Values in the same column with different superscript letters are significantly different (P < 0.05) while values is the same column that share a superscript letter are not significantly different

${\Delta F}_{hom}$: rate of increase of average ${F}_{hom}$ at neutral, QTL and marker loci; ${\Delta F}_{drift}$: rate of increase of average ${F}_{drift}$ at neutral, QTL and marker loci; ${F}_{hom}-{F}_{drift}$: difference between ${F}_{hom}$ and ${F}_{drift}$ at neutral, QTL and marker loci in the last generation of the simulation

**Fig. 1:**
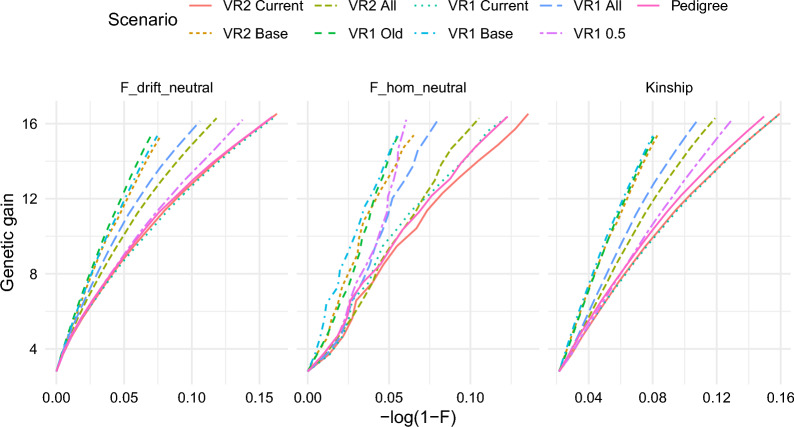
Genetic gain as a function of kinship and inbreeding. Genetic gain in additive genetic standard deviations plotted against inbreeding and kinship when nine different kinship matrices were used for optimum contribution selection for 15 generations. Scenarios are the same as in Table 1. F_drift_neutral: average *F*_*drift*_ at neutral loci; F_hom_neutral: average *F*_*hom*_ at neutral loci; Kinship: average kinship; Genetic gain: Average true breeding value expressed in units of additive genetic standard deviations

Figure 1 shows genetic gain as a function of $f$, ${F}_{drift}$, and ${F}_{hom}$. **VR1 Base** and **VR1 Old** achieved the largest genetic gain per unit $f$, ${F}_{drift}$, and ${F}_{hom}$ and were thus the most efficient, while **VR2 Current** and **VR1 Current** were the least efficient. The ranking of the efficiency of the scenarios, measured as genetic gain per unit of $f$, ${F}_{drift}$, and ${F}_{hom}$, was similar across $f$, ${F}_{drift}$, and ${F}_{hom}$, except for **VR1 0.5**, which resulted in relatively low values of ${F}_{hom}$ compared to ${F}_{drift}$ and $f$.

There were only small differences among the scenarios in terms of the proportion of segregating alleles at the end of the simulation. **VR2 Base** had the highest percentage of segregating QTL, i.e. 84.8%, and **VR1 Base** had the highest percentage of segregating neutral loci, i.e. 84.8%. **VR1 Current** had the fewest segregating QTL and neutral loci, i.e. 83.7% and 83.6%, respectively.

## Discussion

We observed the following major trends in our results: (i) GOCS was more efficient than POCS at achieving genetic gain for a given rate of increase in average $f$, ${F}_{drift}$, and ${F}_{hom}$, (ii) using **Base** or **Old** animals to compute RAF was more efficient at achieving genetic gain for a given rate of increase in average $f$, ${F}_{drift}$, and ${F}_{hom}$ than using **All** animals or **Current** to compute RAF, and (iii) **VR1** resulted in slightly less $\Delta f$ and $\Delta G$ than **VR2**, but the differences were not statistically significant.

In our study, GOCS was able to achieve more $\Delta G$ for a given ${\Delta F}_{drift}$, ${\Delta F}_{hom}$, and $\Delta f$ than **Pedigree**. Several studies of OCS in genetic improvement programs have found that POCS was more efficient than GOCS; Körte [9], Sonesson et al. [19], Henryon et al. [20], Meuwissen et al. [21], and Zhao et al. [34] found that POCS achieved more genetic gain than GOCS for a given rate of true inbreeding, but Clark et al. [35] found that GOCS achieved a similar genetic gain as POCS in a simulation of a dairy cattle population. Maltecca et al. [12] found that the efficiency of GOCS was superior to that of POCS. Henryon et al. [20] argued that the greater efficiency of POCS was because it allowed for changes in allele frequency at QTL, while GOCS, which was implemented using VR1 and base RAF in their study, penalized changes in allele frequency at all markers, thus prevented such changes at QTL in LD with markers, and affected genetic gain. It is possible that the number of selected parents included in the different studies partly explains the different findings on the relative performance of GOCS and POCS. In our study, the number of selected bulls was fixed by design and therefore did not differ between scenarios, but several studies have found that POCS selects more parents than GOCS [9, 20, 21, 34]. However, Clark et al. [35] did not observe this. The different nature of pedigree and genomic kinship estimates may explain the difference in number of selected parents and in relative efficiency of applying OCS with genomic or pedigree-based kinship estimates. Pedigree kinship is an expectation that does not take random segregation of chromosome segments at meiosis into account, while genomic kinship measures realized segregation and should therefore estimate true kinship more accurately. A GRM may be able to discriminate more accurately between selection candidates than a pedigree-based relationship matrix, allowing GOCS to select a set of more diverse animals than POCS, resulting in lower kinship and inbreeding. POCS may compensate by selecting more sires, but our simulation did not allow that because the number of sires was set to 40 in each generation. We suggest that future studies should consider in more detail the number of selected sires when comparing the relative efficiency of different implementations of OCS.

Some OCS studies have focused on applications in conservation programs that do not include genetic improvement. de Cara et al. [36] and Gómez-Romano et al. [37, 38] found that GOCS results in higher genetic diversity than POCS. Morales-González et al. [39] compared different kinship estimators for GOCS using real turbot data in a scheme without selection for genetic improvement. They found that using matrices based on the proportion of shared alleles, shared segments, or excess of shared alleles relative to expected homozygosity under Hardy–Weinberg equilibrium, retained more diversity than VR1, VR2 and Yang’s [40] method. The choice of matrix substantially affected the number of selected parents, with VR1, VR2 and Yang’s method resulting in more animals being selected than other kinship estimators. These simulations did not include selection for genetic gain, but genetic gain is crucial to consider in the application of OCS for commercial breeds. As noted by Henryon et al. [20], genomic and pedigree kinship estimates differ in how much they restrict changes in QTL allele frequencies. Since conservation programs usually do not include any genetic improvement, but aim at maximizing genetic diversity, different kinship estimators may be more appropriate for conservation than for genetic improvement programs.

The benefits of using base RAF are clear in our study. Updating RAF, as implemented in our study either by using all genotyped animals or the most recent generation of genotyped animals, allowed more genetic gain compared to using frequencies of previous generations, but at the cost of substantially higher $\Delta f$, ${\Delta F}_{drift}$, and ${\Delta F}_{hom}$. Our study explicitly tested the use of base and recent RAF at a fixed number of sires and confirmed that using base animals to estimate RAF is a better option than updating the RAF with more recent animals. This was not only true in our scenario when the true RAF were used (**VR1 Base**), but also when bulls selected in the first four generations were used (**VR1 Old**). VanRaden’s estimators of relationship represent the probability of identity-by-descent relative to the base population, from which the RAF are computed [22]. When current RAF are used, the average genomic relationship among animals is close to zero and pairwise relationships can take negative values. Therefore, the interpretation of relationships as a probability is not appropriate and the estimates should be interpreted as correlations, where negative values imply less relationship between individuals than the average relationship [41]. As shown by Wang [41], genomic estimators of kinship were developed using base frequencies as reference. The use of the same sample to estimate both kinship and allele frequencies violates the assumption of independence of these parameters. When the base population is used to estimate RAF, relationships are estimated according to the whole genealogy, but more current RAF estimate relationships relative to the more recent part of the genealogy.

Higher selection pressure was reflected in lower MAF for scenarios using more recent RAF. The tendency of **VR1 0.5** to promote heterozygosity was shown by the relatively high MAF, and low ${F}_{hom}$, but at the cost of substantial drift. This was in line with the results of Meuwissen et al. [21].

Our results suggest that implementation of OCS in practice should estimate RAF using the oldest genotype data available. In dairy cattle, cryo-preserved semen from old bulls is often available and genotyping these animals may be useful to estimate RAF for GOCS. Alternatively, the method of Gengler et al. [42] may be used to estimate gene content of animals for which pedigree is available but biological samples for genotyping are not. Such gene content estimates could then be used to estimate RAF for the base population. However, the usefulness of that approach needs to be studied.

This study used simulated data and looked at long-term effects but applications should also be studied using real data. A study by Eynard et al. [43] used real whole-genome sequence data of Holstein bulls and compared OCS using Yang’s [40] method and the numerator relationship matrix. They found differences in the number of selected animals when there was no restriction on the number of selected animals, a GRM based on Yang’s method with RAF set to 0.5 (which they call a similarity-based-method) selected much fewer animals than Yang’s method. They also found that Yang’s method maintained more genetic diversity measured as preserved variants than VR1 and VR2. When the numbers of selected animals were constrained to 10 or 20, the similarity-based method achieved the highest genetic gain, but Pedigree achieved the highest genetic gain when five animals were selected. To further explore the benefits of OCS in Icelandic Cattle and other populations, selection based on different kinship matrices should be compared using real data.

The simulated population in this study was closed, i.e. no imports were simulated. Admixed populations have to be investigated in a separate study. In an admixed population using GOCS with base RAF estimated from animals that were born before admixture, GOCS would tend to move allele frequencies in the population towards the unadmixed base population. Whether this is desirable depends on the goals of the genetic management of the population.

Additive genetic variance is expected to decrease proportionally to mean kinship in the population [44], but in our study, loss of additive genetic variance ($\Delta {\sigma }_{A}^{2}$) was about four to five times greater than increase in kinship ($\Delta f$), as shown in Table 1. This may be due to the effects of selection on QTL allele frequencies.

### Inbreeding due to drift and homozygosity

We used two estimators, ${F}_{drift}$ and ${F}_{hom}$, to evaluate inbreeding based on drift and homozygosity at markers, QTL, and neutral loci. Measured in this way, ${F}_{drift}$ includes both the effects of random drift and the effects of selection. Selection moves allele frequencies at QTL in a beneficial direction but drift causes random changes at QTL allele frequencies. The former is desired whereas the latter is detrimental for the breeding program. Drift at neutral loci, that do not affect the selected trait, is usually considered undesirable, since such drift can randomly affect other traits that are not part of the current breeding goal. Such drift can randomly change values for traits that are valuable but not selected, and it can also result in loss of genetic variation for future selection [21]. We simulated neutral loci that are in LD with markers and QTL. This method should resemble a real genome in which neutral loci are in LD with marker loci. The patterns of ${F}_{drift}$ and ${F}_{hom}$ were similar across the three types of loci but there were some differences between the kinship matrices. By using **Pedigree** and **Current** RAF, more drift was observed at all types of loci, as measured by ${F}_{drift}$. Using RAF of 0.5 resulted in relatively low ${F}_{hom}$ at the expense of higher ${F}_{drift}$, in line with the finding of Meuwissen et al. [21]. A simulation of a conservation scheme by Morales-González [45] found similar results. In their study [45], a matrix based on Li and Horvitz [46] maintained higher expected heterozygosity than VR2 but at the cost of higher drift, similarly to **VR1 0.5** in our study. Meuwissen et al. [21] observed that VR1 and VR2 resulted in higher ${F}_{hom}$ than ${F}_{drift}$ at neutral loci, that RAF of 0.5 resulted in higher ${F}_{drift}$ than ${F}_{hom}$, and that **Pedigree** resulted in roughly equal ${F}_{hom}$ and ${F}_{drift}$. Conversely, we observed that ${F}_{hom}$ was lower than ${F}_{drift}$ for all scenarios. This was true both for markers with a MAF distribution that resembled commercial SNP chips (the panel *Markers* used for genetic prediction), and for the neutral and QTL loci which had a MAF distribution resembling that of whole-genome sequencing data as reported by Meuwissen et al. [21]. The selection schemes in these two studies were different and may explain the different results. In the study of Meuwissen et al. [21], the performance of full-sibs of the selection candidates was used to train the genomic prediction model. Full-sib families were then created in each generation, in which half of the sibs became selection candidates and the other half test-sibs. In our study, performance was measured on the dams of the selection candidates and the population had a half-sib structure as in dairy cattle breeding. Another difference is the implementation of the OCS computations. Our study optimized contributions so that a fixed number of sires was selected for a target increase of kinship equal to or lower than the target of 0.005, while Meuwissen et al. [21] set a target of exactly 0.005 and did not fix the number of parents. Thus, it is possible that the different results between our study and that of Meuwissen et al. [21] are due to the number of sires being constrained in our study, and also to the sib structure having an effect on the results. According to Robertson [47], heterozygosity will be lower than that expected according to Hardy–Weinberg equilibrium by the proportion $\frac{1}{8M}+\frac{1}{8F}$ where $M$ and $F$ are the numbers of males and females. Thus, in our study, we expected ${F}_{drift}$ to be higher than ${F}_{hom}$ by 0.0031, but this difference was much larger for all scenarios, and we have no explanation for this.

Meuwissen et al. [21] concluded that the choice of kinship matrix for OCS should reflect the purpose of inbreeding control; whether it is to reduce drift, measured by ${F}_{drift}$, or to maximize genetic variance, measured by ${F}_{hom}$. Our results confirm that there are differences in the effects of different kinship matrices on drift and homozygosity. An interesting topic for future research is which part of inbreeding management is more important for a small dairy cattle population, restraining drift or restraining increase in homozygosity.

### Limitations

Our study compared the effects of kinship matrices for a fixed number of sires, an equal use of them and random mating of selected males to females. We did not simulate any selection on the female side and used discrete generations. Although the assumptions of no female selection, of a fixed number of sires, an equal use of sires, and of random mating deviate from the practical situation, we do not believe they affect the results of our study. We believe that the constraints imposed on OCS in our study resemble a realistic situation for a small dairy cattle population. However, the results may not be generalizable to all breeding programs and the effects of kinship matrices should be studied with other breeding program structures. Because the kinship matrices measure kinship differently, selection resulted in different kinship rates across scenarios, which complicates the comparisons. We optimized the genetic contributions so that the target rate of kinship was computed relative to all animals in each generation. This is not possible in practice for GOCS because genotype data are only available for genotyped individuals, which in this case were 6000 females and 2000 bulls. Optimizing genetic contributions relative only to genotyped individuals will result in kinship rates exceeding the target. Thus, in real application, the realized inbreeding rate will exceed the target rate, unless the genotyping of both sexes is random or all animals are genotyped. In real genomic dairy cattle breeding programs, cow genotyping is common, which should provide a relatively unbiased sample of female genotypes, but the genotyping of bulls is only carried out to identify new AI sires. Therefore, selection will cause the genotyped bulls (which are the selection candidates) to be more related on average than the average relationship among all males in the population, resulting in a higher rate of increase in kinship than the target. Breeders can deal with this issue by simply taking into account that realized inbreeding will be higher than the target rate when genotyping of sires is biased. Further studies on OCS should consider how to deal with this issue in real populations. If not all animals are genotyped, a combined genotype and pedigree matrix could be used.

### Recommendations for breeding programs

We recommend that breeding programs resembling the one that is simulated in this study apply OCS using VR1 with RAF that are estimated from old animals, possibly from old artificial insemination bulls. The use of user input RAF is implemented in software such as GMATRIX [26]. Therefore, the implementation of our recommendation involves minimal costs to a genomic breeding program that uses OCS.

## Conclusions

Genomic OCS is preferable to POCS if base animals are used to compute RAF and a fixed number of sires is selected in each generation. Using base animals to compute RAF for GOCS results in less inbreeding per unit of genetic gain than using recent animals to compute RAF. We did not find significant differences in the performance of VR1 and VR2. Previous studies have found contradicting results regarding the relative performance of GOCS and POCS but our study found clear benefits of GOCS. We suggest that further research should compare fixed and varying numbers of parents in OCS schemes. We recommend that for a small genomic dairy cattle breeding program, implementation of OCS should be based on VR1 with RAF estimated from base animals.

## Data Availability

All code and simulated data for this study can be accessed on this github page: https://github.com/EgillG/IcelandicCattleBreedingSchemes.
